# Tumor-reducing effect of the clinically used drug clofazimine in a SCID mouse model of pancreatic ductal adenocarcinoma

**DOI:** 10.18632/oncotarget.11299

**Published:** 2016-08-16

**Authors:** Angela Zaccagnino, Antonella Managò, Luigi Leanza, Artur Gontarewitz, Bernhard Linder, Michele Azzolini, Lucia Biasutto, Mario Zoratti, Roberta Peruzzo, Karen Legler, Anna Trauzold, Holger Kalthoff, Ildiko Szabo

**Affiliations:** ^1^ Institute for Experimental Cancer Research, Medical Faculty, CAU, Kiel, Arnold-Heller-Strasse 3 (Haus 17), Germany; ^2^ Department of Biology, University of Padova, viale G. Colombo 3. Padova, Italy; ^3^ Department of Biomedical Sciences, University of Padova, Italy; ^4^ CNR Institute of Neuroscience, Padova, Italy

**Keywords:** apoptosis, pancreatic ductal adenocarcinoma, potassium channel, clofazimine, orthotopic model

## Abstract

Pancreatic ductal adenocarcinoma (PDAC) represents the most common form of pancreatic cancer with rising incidence in developing countries. Unfortunately, the overall 5-year survival rate is still less than 5%. The most frequent oncogenic mutations in PDAC are loss-of function mutations in p53 and gain-of-function mutations in KRAS. Here we show that clofazimine (Lamprene), a drug already used in the clinic for autoimmune diseases and leprosy, is able to efficiently kill *in vitro* five different PDAC cell lines harboring p53 mutations. We provide evidence that clofazimine induces apoptosis in PDAC cells with an EC_50_ in the μM range via its specific inhibitory action on the potassium channel Kv1.3. Intraperitoneal injection of clofazimine resulted in its accumulation in the pancreas of mice 8 hours after administration. Using an orthotopic PDAC xenotransplantation model in SCID beige mouse, we show that clofazimine significantly and strongly reduced the primary tumor weight. Thus, our work identifies clofazimine as a promising therapeutic agent against PDAC and further highlights ion channels as possible oncological targets.

## INTRODUCTION

Pancreatic ductal adenocarcinoma (PDAC) is one of the most aggressive types of tumors, being the fourth leading cause of cancer mortality. In general, patients are diagnosed at a rather late stage of disease and their life expectancy is at most five years after diagnosis. In most cases, the only valid therapeutic approach is the radical surgical resection of the tumor, which however is feasible only in 20% of the cases, and recurrence of cancer lesions often occurs. Some other patients (30-40%) show unresectable locally advanced pancreatic cancer (LAPC) with a median survival of one year [1]. For the rest of the patients, who manifest metastasis at diagnosis, six months is the expected survival period (for a detailed review see e.g. [2]).

For about 20 years, the only therapeutic option considered valid for the treatment of PDAC has been 5-fluorouracil (5-FU). Although the most widely used chemotherapy drug is gemcitabine, administered alone or in combination with other chemotherapeutics such as 5-FU, capecitabine, platinum analogues and taxane [3], currently FOLFIRINOX (mix of 5-fluorouracil, leucovorin, irinotecan and oxaliplatin) has become a promising first line therapy in patients [4]. The mechanism of action by which the cytotoxic effect is exerted by these molecules is the block of DNA synthesis; therefore considerable side effects occur in the healthy tissues. New molecules with aberrant expression in PDAC tissues have recently emerged as possible alternative targets in pancreatic tumor treatment. Among these are the epidermal growth factor receptor (EGFR) [5], human epidermal growth factor receptor type 2 (HER2) [6] and vascular endothelial growth factor (VEGF) [7]. For all of them a phase II or III clinical trial has been conducted but the improvement of outcome for PDAC patients is not satisfactory. Therefore, new therapeutic approaches are necessary.

During the last decade, ion channels emerged as possible prognostic markers and therapeutic targets against various types of cancer as indicated also by *in vivo* studies in preclinical models [8, 9]. In particular, potassium (K^+^) channels have been shown to be crucially involved in many important physiological processes such as proliferation, migration, angiogenesis, cell volume regulation and apoptosis [10, 11] and to promote cancer progression [12]. The voltage-gated potassium channel Kv1.3 belongs to the *Shaker* family (Kv) and is expressed in different tissues, such as brain, kidney, olfactory bulb, lymphocytes, skeletal muscle, adipose tissue [13]. In healthy individuals Kv1.3 is prevalently expressed mainly in the CNS and in immune cells [14]. Aberrant (mostly high) expression of Kv1.3 has instead been observed in different types of tumors [13, 15], such as melanoma [16], prostate [17], breast [18], B-cell lymphoma [19], chronic lymphocytic leukemia (B-CLL) [20, 21] gastric [22], pancreatic tumor [23] and in lung cancer [24]. Overexpression of Kv1.3, like that of other Kv channels, in cancer cells could give an advantage to cancer cells enhancing tumorigenic processes such as proliferation, cell migration and metastasis [10].

Kv1.3 is expressed and active both in the plasma membrane (PM) and in the inner mitochondrial membrane (IMM) of lymphocytes (mtKv1.3) [25], hippocampal neurons [26] and in various tumor cells [27, 28]. Kv1.3 was located to the cis-Golgi membranes as well [29] and, recently, to the nuclear membrane [30] where it seems to be involved in regulation of transcription. Expression of the channel in the IMM seems to correlate with that in the PM (e.g. [27]), but while the plasma membrane Kv1.3 is required for cell proliferation, the mitochondrial channel regulates apoptosis. At the molecular level, mtKv1.3 is a target of the pro-apoptotic protein Bax, which inhibits the channel via a conserved positive amino acid residue, lysine 128, with a Kv1.3-inhibiting toxin-like mechanism [31, 32]. Bax, or membrane permeant Kv1.3 inhibitors which can reach the mitochondrial channel, block the positive inward (toward the mitochondrial matrix) potassium current, thereby determining a transient hyperpolarization followed by the release of reactive oxygen species (ROS), the opening of the permeability transition pore with consequent loss of mitochondrial membrane potential and release of cytochrome c, finally leading to the activation of apoptotic cascade.

We have previously shown that *in vitro* inhibition of mtKv1.3 using membrane-permeant Kv1.3 inhibitors such as Psora-4, PAP-1 and clofazimine results in apoptosis of various Kv1.3-expressing tumor cells [20, 28, 33]. These drugs were efficient even in the case of poor-prognosis tumor cells lacking Bax/Bak and bearing p53 mutations. Importantly, this effect was observed exclusively with the membrane-permeant inhibitors, indicating the importance of the mtKv1.3 versus PM Kv1.3. Genetic deficiency or siRNA-mediated downregulation of Kv1.3 abrogated the effects of these substances. One of these specific drugs, by targeting mtKv1.3, was able to reduce tumor volume *in vivo* by 90% in a preclinical model of melanoma, without side effects [28]. The drugs also killed primary pathological B cells from chronic lymphocytic leukemia patients, without affecting survival of healthy T cells of the same individuals [20]. The proposed mechanism of action points to a synergistic effect between the altered redox state, characteristic of tumor cells and the ability of the above drugs to induce significant oxidative stress by inhibiting mtKv1.3, leading finally to ROS-induced cell death in the cancer cells, while leaving healthy cells alive.

Clofazimine blocks Kv1.3 channel activity with an EC_50_ of 300 nM, while it is much less effective on other potassium channels of the same Kv family [34]. However, for its apoptosis-inducing action a higher, μM concentration is required, presumably due to the accumulation of the drug in membranes other than the IMM. Clofazimine is a lipophilic riminophenazine antibiotic approved by the FDA and already in clinical use to treat pathologies like leprosy and psoriasis; its antibiotic, immunomodulatory and anti-inflammatory properties make it a versatile drug [35]. In light of the results obtained with clofazimine on B-CLL cells and in the orthotopic melanoma model, and of indications of expression of Kv1.3 in pancreatic cancer tissues, we investigated whether this drug can be used against PDAC in an orthotopic xenograft model.

## RESULTS

### Kv1.3 is expressed in different human PDAC cell lines

**Figure 1:**
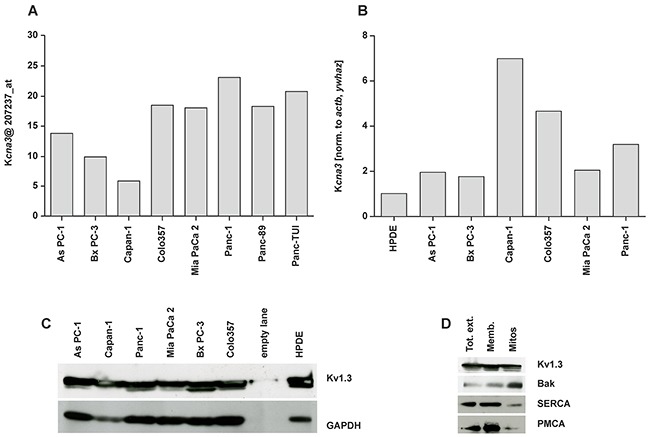
Expression of Kv1.3 potassium channel in different pancreatic ductal adenocarcinoma cell lines **A**. Histogram of the distribution of Kcna3 gene expression in a panel of pancreatic cancer cell lines from Affymetrix U133 Gene Chip. Arbitrary intensity units of the probe ID (207237_at) for Kcna3; the intensity values were normalized with dCHIP2006 software (www.dchip.org) **B**. The relative level of gene expression as determined by quantitative RT-PCR was calculated using qBase_Biogazelle software, which allows a multiple reference genes-normalization and performs inter-run calibration. *Actb* and *Ywhaz* of non-tumoral HPDE cells were set as reference genes (value 1) to normalize the gene expression. **C**. Whole cell extracts (50 μg/lane) from different PDAC cell lines were loaded on SDS-PAGE. Western blot revealed Kv1.3 bands (multiple bands are presumably due to glycosylation according to manufacturer or to degradation products) at around 65 kDa. These bands correlated with Kv1.3 expression since none of the bands were present in a cell line silenced for Kv1.3 (not shown). The same blot was developed with the antibody against GAPDH (45 kDa) as loading control. **D**. Whole cell extract (Tot ext), enriched membranous fraction (Memb) and Percoll-purified mitochondria (mitos) fractions obtained from Colo357 were loaded at equal protein concentration (40 μg/lane). Enrichment in the mitochondrial marker Bak and decrease of the intensity of SERCA (ER marker) and PMCA (PM marker) indicates a higher purity of the mitochondrial preparation. Results in C-D are representative of Western blots from three independent experiments.

Since our purpose was to understand whether clofazimine might be useful to induce apoptosis in PDAC cell lines via inhibition of the mitochondrial Kv1.3 channel, first we checked whether some of the widely used human PDAC lines of various origin express Kv1.3. To this end, we extracted information available in a previously reported Affymetrix U133 GeneChip set [36, 37]. Analysis of the microarray data shows that in 5 out of 8 lines expression of the *Kcna3* gene encoding for Kv1.3 channel was relatively high (Figure 1A). To confirm these data in the cell lines used in our laboratories and to compare Kv1.3 expression with that of a non-tumoral pancreatic ductal epithelial line, we performed quantitative real time PCR using multiple reference genes of the non-tumoral HPDE line for normalization (Figure 1B). HPDE6-E6E7 (H6C7) is a non-tumoral, immortalized human pancreatic ductal epithelial cell line, generated by transformation with human papilloma virus 16 (HPV-16). HPDE does not have the typical characteristics of pathological cells except a very fast growth kinetic. Cultured HPDE cells express low levels of epidermal growth factor receptor (EGFR), erbB2, transforming growth factor (TGF)-alpha, Met/hepatocyte growth factor receptor (HGFR), vascular endothelial growth factor (VEGF), and keratinocyte growth factor (KGF). HPDE was shown to be non-tumorigenic when transplanted into SCID mice [38].

The human tumor lines that were used have different origin and characteristics. All these lines have been characterized in detail in several previous works [39–41] and were found to be mutated in p53. Most of them, with the exception of Bx PC-3, are mutated also in K-RAS [41]. It is of note, that these cell lines, harboring mutation in p53, have been found previously to be largely resistant to standard chemotherapeutics. Mutations in the p53 gene have been described in more than 50% of the patient samples and there is evidence that the p53 network is genetically altered in an even much higher proportion. As PC-1 is a human pancreatic tumor cell line that was derived from the metastatic site, an ascites, of a patient with adenocarcinoma in the head of the pancreas. The As PC-1 nude mouse tumor model shows characteristics similar to those of human PDAC, with abundant mucin production and granular differentiation. Bx PC-3 is a primary human pancreatic tumor cell line from the body of the pancreas of a patient with adenocarcinoma. These cells are not prone to give metastasis and they are poorly differentiated. The Bx PC-3 nude mice tumor model also shows characteristics close to those of human PDAC with mucin production and displays moderately differentiated adenocarcinomas with occasional lymphocytic infiltrations at the tumor peripheries. Colo357 cells originate from a lymph node metastasis from a PDAC patient and display p53 mutation (Trauzold et al, unpublished result). They express low levels of Bcl-xL, are highly metastatic and are moderately differentiated. Capan-1 PDAC line, originally obtained from liver metastasis, is highly resistant to different chemotherapeutic drugs, in particular to 5-FU. Tumor models using Capan-1 show a well-differentiated adenocarcinoma with abundant mucin production. The poorly differentiated Mia PaCa 2 cell line was initially obtained from the body and tail of the pancreas of a PDAC patient, while the metastatic Panc-1 line was isolated from the head of the pancreas. This tumor had invaded the duodenal wall of the patient and expresses a high level of the anti-apoptotic Bcl-xL [42]. To confirm that Kv1.3 was expressed also at protein level, we performed Western blot analysis on whole-cell extracts (Figure 1C), revealing the presence of the channel protein in all cell lines. Finally, in one of these lines, namely in Colo357 which was used for the *in vivo* studies in this paper (see below), we assessed mitochondrial expression of Kv1.3. Purity of the mitochondrial fraction is indicated by the low intensity of PM (PMCA) and ER (SERCA) markers. In contrast, the band intensity of the mitochondrial marker Bak (Figure 1D) is high in the mitochondrial fraction which also contains Kv1.3. Altogether, these results indicate the presence of Kv1.3 in different PDAC cell lines.

### Clofazimine decreases cell survival in PDAC lines harbouring p53 mutations but spares non-tumoral lines

**Figure 2:**
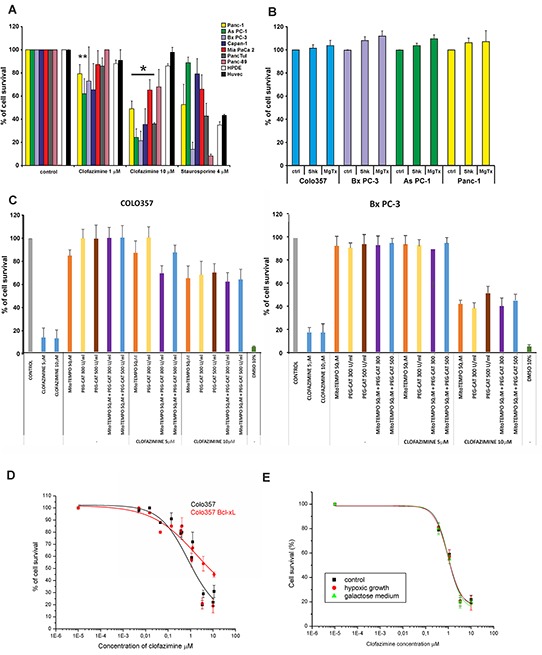
Clofazimine reduces cell survival in PDAC lines with p53 mutation **A**. MTT assay indicates decrease of cell survival at the higher clofazimine concentration applied in seven independent PDAC lines, while two non-tumoral lines were largely resistant to clofazimine treatment. Staurosporine was used as positive control. Statistically significant effects are marked with asterisk (p<0.05). **B**. Lack of effect of membrane-impermeant Kv1.3 inhibitor toxins on cell survival. PDAC lines were treated as in A), with the indicated doses of the toxins. **C**. Colo357 and Bx PC-3 cells were incubated with clofazimine, either without or with pre-treatment of the cells with mitochondria-targeted ROS scavenger (MitoTEMPO) or with membrane-permeant catalase (PEG-CAT). Data are reported as percentage of control cells +/− S.D. (n=4). **D**. Dose-response curve of the effect of clofazimine in wt Colo357 cells and in those stably overexpressing anti-apoptotic Bcl-xL. Higher than 10 μM clofazimine was not used due to drug precipitation above this concentration. **E**. As in B) in cells grown under hypoxic conditions or following culturing in galactose. In A, D and E the results shown are mean values ±SD (n= from 3 to 11). Fitting was obtained using the Origin 7.5 program set.

Next, we studied the effect of clofazimine in the above PDAC lines in a previously used setting to induce apoptosis. Figure 2A shows that while HPDE and the human umbilical vein endothelial cells HUVEC were resistant to 10 μM clofazimine, basically all PDAC lines, even those that are resistant to 4 μM Staurosporine (PANC-1, AsPC-1, Capan-1 and Mia PaCa 2), responded to this concentration of clofazimine with a significant reduction of cell survival. Membrane impermeant toxins (Mgtx and Shk) act prevalently on the PM-Kv1.3 and, as expected, were unable to reduce cell survival in different PDAC lines (Figure 2B). In accordance with the proposed mechanism of action, pre-treatment of Colo357 and Bx PC-3 cells with membrane-permeant catalase and/or a mitochondrially targeted ROS scavenger (MitoTEMPO) substantially decreased the cell-survival reducing effect of clofazimine (Figure 2C). This decrease took place to different extents in the two lines, suggesting that Bx PC-3 lines display a higher basal ROS level. These results suggest that apoptosis induced by clofazimine does not depend exclusively on the level of Kv1.3 expression but also on the basal redox state which might slightly differ between the different cell lines used in this study. For wt Colo357 cells, the EC_50_ for clofazimine-induced cell survival decrease was 1.5 μM. Although this value increased to 6 μM in Bcl-xL-overexpressing cells [43], there was still a significant impairment of cell survival at the highest concentration of clofazimine (Figure 2D). This result is in agreement with the action of clofazimine on the IMM-located mtKv1.3, i.e. downstream and largely independent of the OMM-located Bcl-2 family members. In addition, we tested whether hypoxic conditions, typically found in solid tumors and also in PDAC [44], affect the apoptosis-inducing ability of the drug, which was not the case. Metabolic reprogramming from mitochondrial aerobic respiration to aerobic glycolysis is a hallmark of many types of cancer. Galactose is not used efficiently as glycolytic substrate, therefore the cells need to switch their metabolism to produce all of their energy from oxidative phosphorylation for survival. The switch of the medium did not impact on the *in vitro* effect of clofazimine (Figure 2E).

**Figure 3:**
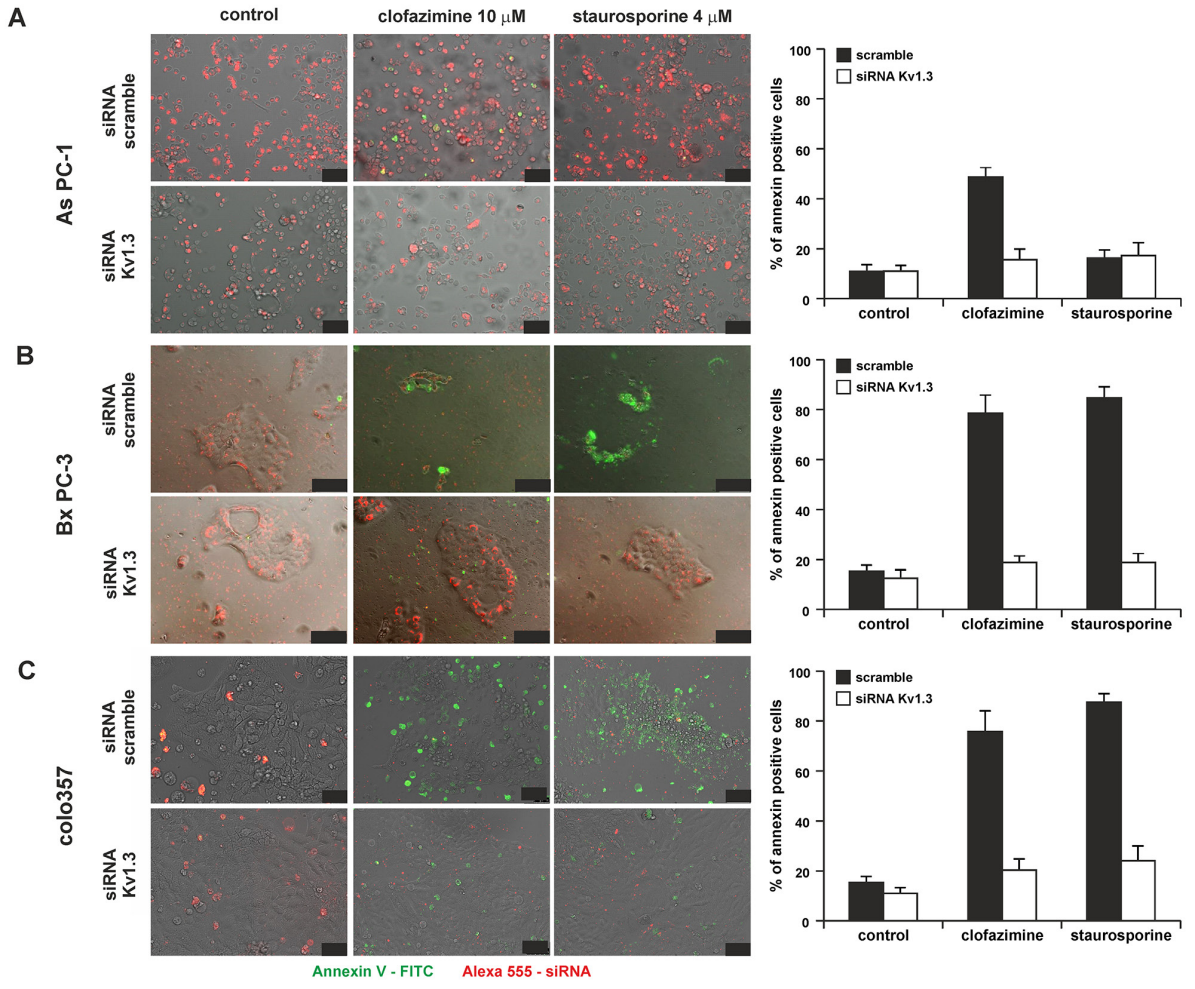
Clofazimine induces apoptosis in PDAC lines in a Kv1.3-dependent manner Effect of clofazimine on the As PC-1 **A**. Bx PC-3 **B**. and Colo357 **C**. PDAC lines following transfection with scrambled RNA or siRNA against Kv1.3. Bars correspond to 50, 100 and 100 μm. Transfection efficiency is indicated by the red signal, since the siRNAs were labeled with Alexa568. Apoptotic cells are visualized by binding of FITC-labeled Annexin to phosphatidylserine (green signal). Staurosporine was used as positive control and has previously been shown to induce apoptosis in Kv1.3-expressing cells [31]. The images are representative of three independent replicas giving the same results. Size bars correspond to 50, 100 and 50 μm in A), B) and C) respectively. Right panels report % of Annexin-positive apoptotic cells from 3 independent images.

In order to prove that reduction of cell survival correlated with increased apoptosis and that clofazimine induced cell death via its action on Kv1.3, we analysed Annexin binding in Bx PC-3 and As PC-1 cells transiently transfected either with scrambled RNA or with siRNA against Kv1.3 (Figure 3A and 3B). In both cell lines clofazimine-induced apoptosis and Annexin binding was evident after treatment with 10 μM clofazimine, even without addition of MDR inhibitors (in agreement with Figure 2A) when Kv1.3 is expressed, but upon silencing of the channel expression the drug was not effective anymore. Staurosporine was used as positive control, and in agreement with data of Figure 2A, this assay confirmed the resistance of As PC-1 cells to this classical apoptosis-inducer, as well as the sensitivity of Bx PC-3 cells. Similar results were obtained also with Colo357 cells (Figure 3C).

### Clofazimine reduces tumor weight

**Figure 4:**
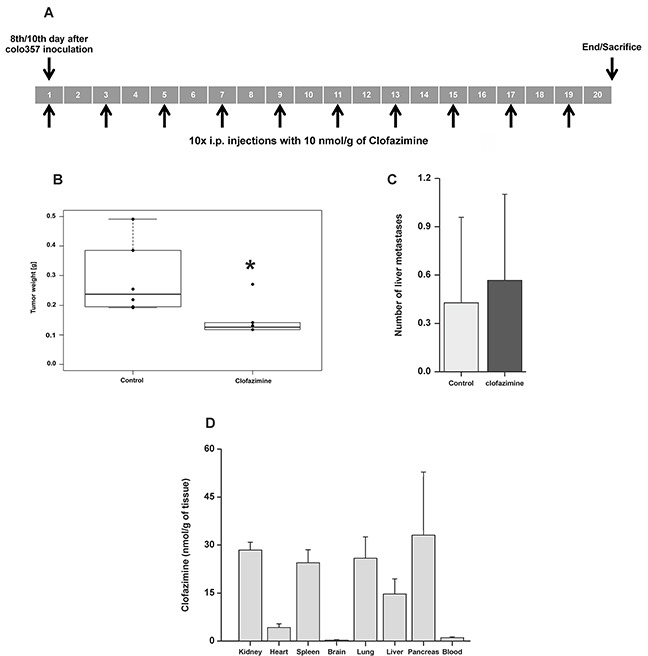
Reduction of tumor weight by clofazimine treatment in a SCID orthotopic PDAC model **A**. SCID beige mice were treated with 5μg/g (10 nmol/g) body weight of clofazimine using the protocol indicated in the scheme. **B**. Tumor weight was determined for the 6 mice of each group at the end of the treatment as in (A). Statistical analysis shows significant difference (p<0.05) between the two groups. **C**. Mean number of liver metastases ± SD for all 6 mice. The difference is not statistically significant (p>0.05). **D**. Tissue accumulation of clofazimine determined as described in the Materials and Methods section.

To further prove the relevance of our findings *in vivo*, we treated severe combined immunodeficient (SCID) beige mice bearing orthotopically xenotransplanted human Colo357 cells with intraperitoneally (i.p.) injected clofazimine ten times, starting 10 days after tumor cell inoculation and continued every second day (Figure 4A). For the treatment regimen we followed a previously established inoculation protocol for Colo357 [45] and then analyzed tumor growth and metastasis. A statistically significant reduction by more than 50% of tumor weight occurred in the clofazimine-treated mice compared to the control mice treated with the solvent (Figure 4B). Instead, no difference in the number of liver metastasis occurred in the clofazimine-treated animals with respect to controls (Figure 4C). In order to prove that clofazimine reaches the pancreatic tissue, we determined the clofazimine concentration in various tissues. Following i.p. administration of a single dose of clofazimine, it was found to be readily absorbed. Low concentrations of the drug were detected in blood. HPLC−UV−ESI/MS analysis of whole tissues showed that clofazimine was basically absent in the brain indicating that it does not readily cross the blood brain barrier and was accumulated only to a low extent in the heart. In the pancreas, kidney, liver and spleen, relatively high concentrations of clofazimine were found (Figure 4D).

**Figure 5:**
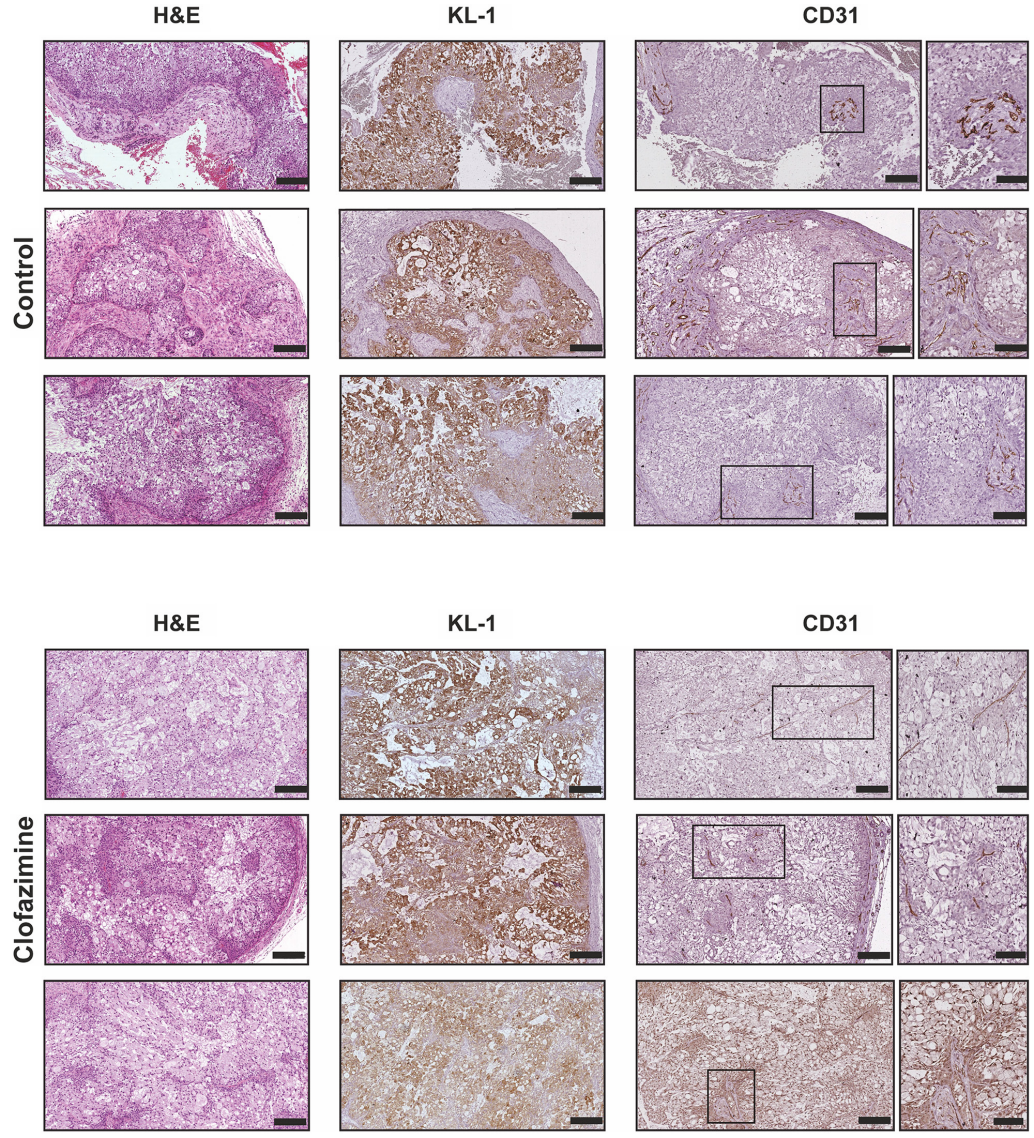
Immunohistochemical analysis of PDAC tissues reveal no gross alterations of tumor structure and angiogenesis upon clofazimine treatment Histopathological characterization of the orthotopic pancreatic cancer model derived from Colo357 cell line. Formalin-fixed and paraffin embedded specimens derived from primary tumour were sectioned and evaluated for the tumour markers pan-cytokeratin1 (KL1) and platelet endothelial cell adhesion molecule (CD31), in order to evaluate the tumour area and the relative angiogenesis, respectively. The morphology of the tissues was examined via Hematoxylin and eosin (H&E) staining. For the CD31 analysis, the framed parts of the sections were scanned at higher magnification (20-fold) to highlight the structure of the CD31-positive cells. Bars correspond to 200 μm and 100 μm for images with higher magnification. Three representative images are shown for each staining from two different mice/condition.

**Figure 6:**
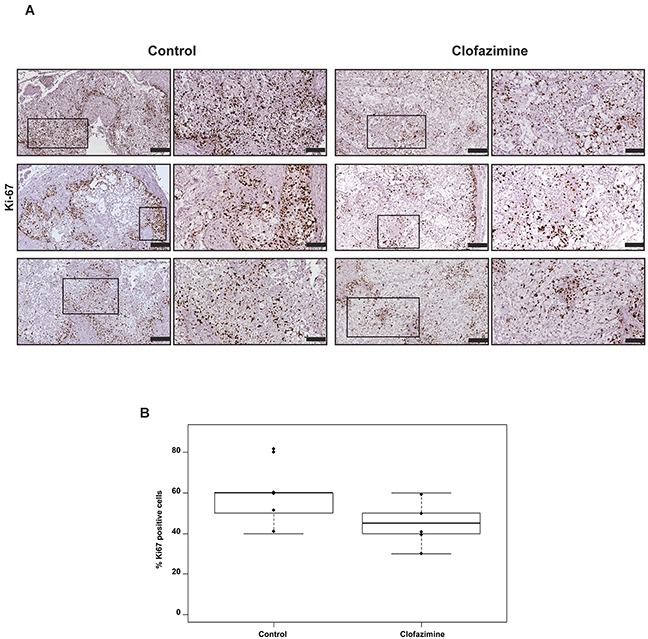
Clofazimine treatment slightly reduced the number of Ki67 positive cells in orthotopic pancreatic tumour model derived from Colo357 cell line Formalin-fixed and paraffin embedded specimens derived from primary tumour were sectioned and stained for Ki67 with SP6-clone antibody (brown stain). The tumour areas positive for KL1 (see Figure 5) were further analysed. **A**. Comparison of Ki67 expression between clofazimine treatment and control group (three representative samples).The framed regions are shown at higher magnification to depict the differences in the Ki67 abundance between the treatment and the control group. Bars correspond to 200 μm and 100 μm for images with higher magnification. **B**. Box-plot showing the evaluation of the number of Ki67-positive cells per 100 tumour cells. The percentage of Ki67-positive cells indicates the proliferative capacity of the cells. Samples from all six mice/group were evaluated revealing a statistically non- significant difference (p-value=0.1433).

Next, we performed an immunohistochemical staining in order to characterize the orthotopic tumor tissues. The morphology of the tissues was examined via hematoxylin and eosin (H&E) staining (Figure 5). We further analysed the tumor area, proliferation and angiogenesis via staining of the tissue with antibodies against KL1, CD31 (Figure 5) and Ki67 (Figure 6), respectively. The anti-human KL1 antibody recognizes many-cytokeratins and binds to cells of epithelial origin. Therefore, it stains neoplastic cells of human and epithelial origin (i.e. of Colo357) and permits the detection of tumor area and to distinguish the human PDAC-derived tissue from the murine pancreas. Anti-CD31 recognizes the platelet endothelial cell adhesion molecule (CD31), a protein which is constitutively expressed on the surface of embryonic and endothelial cells (also monocytes and neutrophils) and represents a marker of angiogenesis. These markers highlighted the presence of larger mucinous lobules in the clofazimine-treated pancreas tissues, but only minor differences regarding tumor structure and angiogenesis were observed between the control and drug-treated groups. Anti-Ki67 recognizes a nuclear protein which is highly expressed in proliferating cells, in particular during late G1-S-M and G2 phases of the cell cycle. Quiescent cells (G0 phase) are negative for this protein. Evaluation of the marker Ki67 showed a reduction in proliferation by only 13% in the Clofazimine treated group (Figure 6A and 6B).

## DISCUSSION

In the present study we report for the first time that clofazimine, a drug already used in the clinic is able to substantially reduce pancreatic tumor growth in an orthotopic ductal pancreas adenocarcinoma model. Clofazimine is an FDA-approved anti-mycobacterial agent recommended by the World Health Organization as part of the standard treatment of leprosy [35]. The use of drugs that have been developed for one disorder and ‘repositioning’ them to tackle another pathology is an increasingly important strategy for researchers in industry and academia [46]. Clofazimine has been in clinical use since the 1960s and is well-tolerated, although bioaccumulation of the drug can lead to visible (but reversible) changes in skin pigmentation. It possesses anti-inflammatory and immunosuppressive activities, therefore it is used in various cutaneous, non-microbial, and chronic inflammatory disorders. In addition, clofazimine has recently been reported to exert potent antifungal activity by inducing a PKC1-dependent stress [47]. More recent studies suggested an expansion of the clinical use of clofazimine to non-cutaneous inflammatory disorders such as multiple sclerosis, rheumatoid arthritis, and type I diabetes mellitus [35]. Despite its known anti-inflammatory activity, the molecular and cellular mechanisms that underlie this property of clofazimine have not been fully elucidated.

We have previously shown that clofazimine is able to induce apoptosis both in primary human tumor cells and *in vivo*, in an orthotopic melanoma model. Inhibition of mtKv1.3 increases mitochondrial ROS production. When pathological B lymphocytes [20] and PDAC cells (Figure 2C) were pre-treated with membrane-permeant ROS scavengers, mtKv1.3 inhibitors failed to induce apoptosis. These experiments suggest that mtKv1.3 inhibition-induced ROS release is necessary for triggering programmed cell death. Since an elevated basal ROS level is known to occur in cancer cells [48, 49], we propose that the clofazimine-induced ROS release drives PDACs over a critical threshold of oxidative stress, causing cell death [50, 51]. In contrast, oxidative stress in healthy cells would not reach the critical threshold, accounting for the cancer-selective action of clofazimine. Beside its action on mtKv1.3, clofazimine inhibits the PM Kv1.3, known to be required for cell proliferation at least in some cell types [14, 24]. Thus, *a priori*, clofazimine might reduce tumor progression by decreasing proliferation. However the slight reduction of the Ki67 index in the examined tumor tissues suggests that tumor reduction was due to the apoptosis-inducing effect of the applied drug.

In our studies we obtained evidence that clofazimine induced PDAC apoptosis prevalently due to its action on the mitochondrial channel, since membrane-impermeant drugs inhibiting only the PM Kv1.3 did not trigger cell death. Mitochondrial potassium channels are known to importantly impact respiration and bioenergetic efficiency of these organelles and as a consequence, ATP production and cell fate [52]. Two observations indicate that mitochondrial function is required for PDAC tumors to develop and grow. First, metformin, an anti-diabetic drug with multiple effects inhibits oxidative phosphorylation (by inhibiting mitochondrial complex I). Metformin exposure has been shown to significantly decrease mitochondrial transmembrane potential, to increase mitochondrial ROS production [53] and to impair PDAC proliferation. Second, a recent study identified a subpopulation of PDACs which was able to survive oncogene ablation and was responsible for tumor relapse. This subpopulation relies on oxidative phosphorylation rather than on glycolysis for cellular energetics and survival. Accordingly, these cells showed high sensitivity to oligomycin, an inhibitor of oxidative phosphorylation. Oligomycin was shown to inhibit tumor recurrence and to increase overall survival rate *in vivo* [54]. Thus, alteration of mitochondrial function, e.g. via modulation of ion fluxes indeed can represent a valid strategy against PDAC.

It must be however mentioned that clofazimine, similarly to metformin is a drug with multiple targets and additional mechanisms accounting for its anti-tumoral effect must be taken into account. For example, clofazimine has recently been identified in a large-scale screening as an inhibitor of Wnt signaling [55], the pathway playing an important role in PDAC tumor initiation and progression [56, 57]. Clofazimine was shown to decrease proliferation of HTB19 triple-negative breast cancer cells whose growth is Wnt pathway-dependent. The authors also reported that clofazimine exerted a significantly smaller effect on non-cancerous mammary epithelial cells (HMEC) than on HTB19 cells, providing thus a further case of selective action on tumoral versus non-tumoral cells [55]. In addition, clofazimine has been shown to exert an inhibitory action on ABCB1/MDR1/P-glycoproteins (Pgp) [58], although this aspect is particularly relevant when applied together with chemotherapeutics to chemo-resistant cells (e.g. [59]). Finally, clofazimine has been shown via bioinformatic screening to have the potential to inhibit p53–MDM2 interaction leading in turn to stabilization and activation of the tumor suppressor p53 [60, 61]. However, in our experimental setting clofazimine induced cell death independently of the p53 status in several cell lines. This finding is particularly relevant given that most PDACs display p53 mutation.

Independently of the exact mechanism of action, in our experiments clofazimine substantially reduced PDAC tumor weight. This ability of clofazimine is in agreement with previous *in vivo* studies obtained in other types of cancer such as hepatocellular carcinoma [62] and melanoma [28]. In those studies the effect of the drug on metastasis was not assessed. The present report suggests that clofazimine might be taken into consideration for reduction of the primary tumor site only, since it did not reduce liver metastasis. The question arises why clofazimine was less effective in the PDAC model with respect to e.g. the melanoma model. We hypothesize that this result may reflect the well-known difficulty of drugs in general to reach PDAC tissues due to the stromal barrier, i.e. a dense extracellular matrix composed of hyaluronic acid, smooth muscle actin and collagen fibers [63, 64]. At the dosages used for the leprosy treatment, with oral administration, the plasma concentrations of the drug reaches 0.5–1 μM [65]. However, a study on tissue concentrations found clofazimine ranging from 0.2 mg/g to 1.5 mg/g (approximately up to 500 μM) in the different organs of a human patient (a value of 0.4 mg/g was obtained for pancreas) [66], which is in agreement with the finding that the drug is generally harmless for normal tissues of the organism. While in the present study a relatively high concentration of clofazimine was found in the pancreas of control animals, it is plausible to suppose that an analogous level could not be reached in PDAC pancreas because of the stromal barrier. Indeed, in a representative experiment with PDAC tumor tissue, we found 3-fold less clofazimine than in the pancreas of healthy mouse (not shown). Furthermore, clofazimine is a rather insoluble molecule: indeed it has been shown to form crystals in the kidney and, during long-term oral administration, to massively accumulate in macrophages, where it forms insoluble, intracellular crystal-like drug inclusions (CLDIs). Recently, it has been reported that *in vitro*, the dissolved fraction of clofazimine was cytotoxic because it depolarized mitochondria and induced apoptosis in macrophages [67] while *in vivo*, CLDIs did not induce macrophage apoptosis. Instead, CLDIs altered immune signaling response pathways downstream of Toll-like receptor (TLR) ligation, leading to decreased NF-κB activation and tumor necrosis factor alpha (TNFα) production and to enhanced interleukin-1 receptor antagonist (IL-1RA) production. Thus, the authors concluded that *in vivo*, macrophages detoxify soluble clofazimine by sequestering it in a biocompatible, insoluble form and this may contribute to the anti-inflammatory activity of the drug. Interestingly, we have recently demonstrated the crucial role of TNFα in the malignancy of PDAC. We found that TNFα strongly increased invasiveness of Colo357 cells *in vitro* and dramatically enhanced tumor growth and metastasis *in vivo* [68]. Furthermore, in severe combined immunodeficient mice with orthotopically growing PDAC tumors, the human-specific anti-TNF antibody infliximab reduced tumor growth and metastasis by about 30% and 50%, respectively. Thus, it is possible that the observed tumor-reducing effect of clofazimine is due to multiple factors, including apoptosis induction of PDAC cells but also to an indirect effect via modulation of macrophage function even in SCID mice. While clofazamine affects tumor growth in the SCID mice, in animals with functional immunity, a priori, Kv1.3 inhibition might adversely alter the function of the immune system [14], leading to a diminished anti-cancer immunity. In our previous study, clofazimine treatment *in vivo* did not cause alteration of blood parameters, but anti-cancer immunity was not investigated [20]. Therefore, this issue needs to be addressed in future work.

In conclusion, our work provides evidence that clofazimine might be a useful agent against PDAC, possibly in combination with currently used chemotherapeutics or newly identified PDAC growth inhibitors such as infliximab. This possibility will have to be addressed in future studies. In addition, combination with other drugs targeting ion channels and/or ion transporters might be a strategy worth pursuing. In fact, thanks to excellent recent works, several proteins mediating transmembrane flux of ions have been identified as possible therapeutic targets in PDAC. For example, hERG1 potassium channels have been shown to drive PDAC tumor malignancy [69], the neurotransmitter GABA was identified as a promising agent for the prevention of PDAC in individuals at risk due to chronic alcohol consumption [70], the transient receptor potential melastatin-related 7 channel was found to regulate tumor migration [71], the store-operated calcium channels (SOCs) were shown to contribute to PDAC apoptosis resistance [72] while the P2×7 receptor regulates cell survival, migration and invasion [73]. Ion transporters which regulate pH are also relevant in this context [74, 75] - e.g. we have recently shown that the specific sodium-proton exchanger NHE1 inhibitor cariporide reduced both three-dimensional growth and invasion and synergistically sensitized these behaviours to low doses of erlotinib [76]. Thus, in an ideal situation, a combined therapy bypassing basic mechanisms responsible for cancer progression and chemoresistance e.g. [77] might be particularly useful.

## MATERIALS AND METHODS

### Cell lines and cell culture

A panel of pancreatic cancer cell lines representing different phases of tumor progression were used. As PC-1, Bx PC-3, Capan-1, Mia PaCa-2 and Panc-1 were provided by ATCC. As PC-1 and Bx PC-3 were cultured in RPMI-1640 (Gibco/Life Technologies, Darmstadt, Germany) supplemented with 10% fetal bovine serum “GOLD” (FBS “GOLD”, PAA Laboratories/GE Healthcare Life Sciences), 1mM GlutaMAX and 1 mM sodium pyruvate (LifeTechnologies, Darmstadt, Germany). Mia PaCa-2 and Panc-1 were cultured in DMEM (4.5 g/l D-glucose) supplemented with 10% FBS “GOLD”, 1mM GlutaMAX and 1 mM sodium pyruvate. Capan-1 cells were grown in IMEM supplemented with 20% FBS “GOLD”, 1mM GlutaMAX and 1 mM sodium pyruvate. Human cell line of metastatic pancreas adenocarcinoma, Colo357, was obtained from Dr. R. Morgan (Denver, CO) [78]. The cells were cultured in a complete growth medium composed of RPMI-1640, 10% FCS (PAN-Biotech, Aidenbach, Germany), 1 mM GlutaMAX and 1 mM sodium pyruvate. The HPV16-E6E7 - immortalized human pancreatic duct epithelial cells (HPDE), kindly provided by Dr. Ming-Sound Tsao (Ontario Cancer Institute, Toronto, Ontario, Canada) [79] were used as a model for benign pancreatic ductal epithelium. The complete growth HPDE-medium was mixed with 50% RPMI 1640, supplemented with 10% FCS and 1mM GlutaMAX and 50% keratinocyte medium SFM (Gibco) supplemented with 0.025% bovine pituitary extract, 2.5 μg l^−1^ epidermal growth factor (Gibco). All cells were cultivated and maintained at 37°C in a humid water-saturated atmosphere with 5% CO_2_. All cell lines were genetically monitored by finger printing. Colo357, As PC-1, Bx PC-3, Capan-1, Mia PaCa-2 and Panc-1 were approved by IonTrac consortium.

### Western blotting

Membrane enriched fraction proteins from different Pancreatic cell lines were obtained as previously reported [80]. Briefly, cells were washed in PBS and then resuspended in 300 μL of TES buffer (100 mM TES + 1 M sucrose + 100 mM EGTA + 1X cocktail protease inhibitors) and lysed by an electric pestle (Kontes, Sigma Aldrich) for 2 min on ice. Unbroken cells were separated by centrifugation at 500 g for 10 min at 4°C. The soluble cytosolic fraction was separated from the membrane-enriched fraction by centrifugation at 19,000 g for 10 min at 4°C. The pelleted membranes were suspended in TES buffer and separated by SDS-PAGE in a 10% polyacrylamide gel containing 6 M Urea. To enhance protein separation, samples were solubilized for 1 h at RT in Sample Buffer (30% Glycerol + 125 mM Tris/HCl pH 6.8 + 9% SDS + 0.1 M DTT + Bromophenol blue). Protein concentration was determined using the BCA method in a 96 well plate (200 μL total volume for each well) incubating at 37°C in the dark for 30 min. Absorbance at 540 nm was measured by a Packard Spectra Count 96 well plate reader. After separation by electrophoresis, gels were blotted overnight at 4°C onto Polyvinylidene fluoride (PVDF) membranes. After blocking with a 10% solution of defatted milk, the membranes were incubated overnight at 4°C with the following primary antibodies : anti-Kv1.3 (1:200, rabbit polyclonal, Alamone Labs APC-101); anti-GAPDH (1:1000, mouse monoclonal, Millipore MAB374). After washing, the membranes were developed using corresponding anti-mouse or anti-rabbit secondary antibodies (Calbiochem). Antibody signal was detected with enhanced chemiluminescence substrate (SuperSignal West Pico Chemiluminescent Substrate, Thermo Scientific).

### MTT assay

To determine cell growth/viability in PDAC cells, we employed the tetrazolium reduction (MTT) assay. To this end, 10,000/well PDAC cells were seeded in standard 96-well plates and allowed to grow in the medium normally used for each line (200 μl) for 24 h. The growth medium was then replaced with fresh phenol red-free medium. Four wells were used for each condition. After 24 h incubation with the indicated drugs 10% CellTiter 96 AQUEOUS One solution (Promega) was added to each well. After 1-3 h of colour development at 37°C, absorbance at 490 nm was measured using a Packard Spectra Count 96-well plate reader as in [78]. Anaerobiosis was obtained by reducing oxygen percentage to less than 1% by inflating nitrogen in a modular incubator chamber (Billups-Rothemberg, USA). Metabolism was altered growing the cells (seeded 3,000/well) for three days in a DMEM lacking glucose but supplemented with galactose, before treatment with the indicated drugs. For experiments with ROS scavangers, 7,500 cells (both Colo357 and Bx PC-3) were seeded in 96 well plates in 200 μL of medium. After 24 h the cells were pre-incubated for 1 h with ROS scavengers, e.g. MitoTEMPO and PEG-Catalase either alone or in combination, using different concentrations as indicated in the figure. Afterwards, cells were treated with 5 or 10 μM clofazimine for further 24 hs. After incubation, 20 μL of MTT solution (Cell titer Aqueous solution, Promega) was added in each well and formazan formation was determined by measuring absorbance at 490 nm with an Infinite 200 Pro NanoQuant plate reader (Tecan).

### siRNA

The sequences for the siRNA targeting human Kv1.3 were coupled to Alexa Fluo 555 (Qiagen). 80,000 cells/well (Colo357, Bx PC-3, As PC-1) were seeded into a 12 well plate in 1 mL of the growth medium. After 24 h cells were transiently transfected with 2 μg siRNA/well using Lipofectamine 2000 as suggested by the supplier. After 48 h from transfection, cells were treated for 24 h more with the drugs, as indicated. Cell death, evaluated by the binding of FITC-labelled Annexin V, as well as siRNA transfection were determined using a DMI4000 Leica fluorescence microscope.

### Purification of mitochondria

Mitochondria from Colo357 cells were purified by differential centrifugation as in [28]. Briefly, approximately 80% confluent cells from eight 150-cm^2^ flasks were washed once with PBS, detached by gentle scraping and spun down in a table centrifuge at room temperature. The pellet was resuspended in sucrose/N-[81]-2-aminoethanesulfonic acid (TES) buffer (300 mM sucrose, 10 mM TES, 0.5 mM EGTA, pH 7.4). After standing for 30 min on ice, cells were lysed in a Dounce homogenizer, and the lysate was centrifuged at 600 x g for 10 min at 4°C. The pellet was again processed in the same way to maximize recovery. The combined supernatants were centrifuged once at 600 x g, and the pellet was discarded. The mitochondria-containing supernatant from the last step was centrifuged at 8,000 x g for 10 min at 4°C. The pellet was gently homogenized and suspended in a small volume of TES buffer. A further purification was obtained by centrifugation (8,500 x g, 10min, 4°C) on a discontinuous Percoll gradient (60, 30 and 18% Percoll in TES buffer). The floating material was discarded, and the fraction at the lower interface was collected and washed three times by centrifugation at 19,000 x g for 5 min. The final pellet was resuspended in TES buffer.

### Gene expression analysis

Kv1.3 gene (*Kcna3*) expression was analyzed in a transcriptome microarray U133 A/B Affimetrix GeneChip set, including microdissected freshly frozen tissues specimen from pancreatic cancer and normal pancreatic tissue, a collection of malignant pancreatic and benign stromal cell llines, according to previously published data set [37]. It consisted of more than 44,000 probes sets detecting 33,000 genes and ESTs. The samples comprised microdissected freshly frozen tissue from PDAC and normal pancreatic tissue. Additionally, a panel of malignant pancreatic cell lines (Capan-2, Bx PC-3, Capan-1, As pc-1, COLO-357, Mia PaCa 2, Panc-1, Panc-89, PT-45, Panc-TUI) and benign stromal cell lines (Kif5, F13 and immortalized primary stellate cells) were also included. The microarray data processing was performed as previously reported [37]. We extracted the expression value of the Kv1.3 in the different pancreatic malignant cell lines with a special emphasis on the lines that were used in the current study.

### Quantitative real time PCR

Total RNA was isolated from culture cells 24 hours after seeding. The total RNA was purified using peqGOLD Total RNA kit and treated with DNase I (PEQLAB Biotechnology GmbH) for removing residual DNA. RNA concentration was measured using a Nanodrop spectrophotometer (PEQLAB Biotechnology GmbH) and the quality of the extracted RNA was checked on 1% agarose gel. 1 μg of RNA was reverse transcribed into cDNA with Maxima First Strand cDNA Synthesis kit (Thermo Fisher Scientific). Real-time PCR was performed in a total volume of 20 μl using 50 ng of the first-strand cDNA synthesis mixture as template. The assay was done with Double-dye (Taqman technology) probes; the exon-exon spanning primers sequence for *Kcna3* (Kv1.3) was supplied by Primerdesign Ltd (Southampton, United Kingdom). The amplification reaction was run in a StepOnePlus Real-time machine according to the follow conditions: Taqman enzyme activation at 95°C, 10 min; denaturation at 95°C, 15 sec, annealing at 50°C, 30 sec; extension at 72°C, 15 sec (fifty cycles). The relative quantification of gene expression was calculated with the help of qBase Browser (Biogazelle NV, Zwijnaarde, Belgium) by applying the comparative C_T_ method with a multiple reference gene normalization. *Actb* and *Ywhaz* were set as reference genes to normalize gene expression; HPDE cells were used as reference sample to determine *Kcna3* relative expression (fold change) in the panel of pancreatic cancer cell lines.

### Laboratory animals

Four weeks old females SCID beige (C.B.-17.Cg-Prkdcscid Lystbb/Crl) mice weighting 14-19 g were obtained from Charles River (Sulzfeld, Germany). They were housed in a sterile environment and allowed to acclimatize for one week. The animal experiments and care were carried out in accordance with the guidelines of institutional authorities and approved by local authorities (number V312-7224.121-7(123-10/11)).

### Orthotopic xenograft of human adenocarcinoma cells and treatment

The orthotopic injection was performed as previously described [82]. Human metastatic pancreas adenocarcinoma cells Colo357 were detached with Accutase solution (PAA Laboratories GmbH), resuspended at the concentration of 10^6^ cells ml^−1^ in 25 μl of Matrigel (BD-Biosciences) and stored on ice. After median laparotomy, 25 μl of cell suspension were injected in the tail of the pancreas. For the therapy, the animals were randomly designated to the treatment procedure: (1) no treatment, (2) clofazimine treatment. The therapy was initiated ten days after tumor inoculation and spanned 20 days. Clofazimine was freshly dissolved at [2.5 mM] in DMSO and sterile water for injection (Ampuwa, Fresenius-Kabi, Germany). For the therapy, it was administered in a dose of 5μg/g (10 nmol/g) intraperitoneally. The control group was treated with a solution containing DMSO and physiological saline buffer. All animals were examined daily for general signs of distress and complications. Thirty days after cell inoculation, the animals were sacrificed and tumor weight was determined after blood removal. Thereafter, the tumors were cut into two equal parts, which were directly either frozen in liquid nitrogen or formalin-fixed and later paraffin-embedded (FFPE). The mice were carefully inspected for any macroscopic metastases in liver, spleen, and mesenterium. Furthermore, several organs were embedded in paraffin for histological staining and analysis.

### Immunohistochemistry

Primary tumor samples from paraffin-embedded tissue were cut into 3μm sections and mounted onto slides. The specimen were tested for pan-cytokeratin (KL1, Beckman Coulter, mouse monoclonal 1:1500), Ki-67 (SP6, ThermoScientific, rabbit monoclonal 1:300) and CD31 (SZ31, Dianova, anti-mousemonoclonal 1:30). The immunohistochemistry (IHC) was carried out according the following procedure: de-paraffinization with xylene, rehydration in descending concentrations of ethanol, followed by heat-induced epitope retrieval with sodium citrate buffer (pH 6.0). Washing steps were performed at room temperature with tris-buffered saline (TBS); to avoid non-specific binding of the antibody to the tissues, all specimen were incubated with POD (Peroxidase-blocking solution, Dako) for fifteen minutes. The primary diluted antibodies (antibody diluent, DCS LabLine, Hamburg, Germany) were applied and the samples were kept in a humidified chamber for one hour. For detection, the tissues were incubated with HPR-conjugate anti-rabbit/mouse (Dako) and Mouse-PO anti-rat for thirty minutes; subsequently, the substrate chromogen-DAB (Dako) was added and the staining developed for five minutes. Finally, the samples were counterstained with Hematoxylin (Dako) and dehydrated with ascending concentrations of ethanol and rinsed in xylene. To test the proliferation activity of the tumors in both control and clofazimine-treated samples, the Ki-67 nuclear staining was evaluated by an experienced pathologist who performed the Ki-67 % scoring. Briefly, for each tumor sample the areas with a positive staining (hot spot) were selected. In the selected field, the cells showing nuclei with weak intensity of the staining were regarded as Ki-67 positive cells and counted at 40X magnification. Finally, the Ki-67 % scoring was determined as number of positive cells compared to the total number of nuclei in the corresponding area. Slides were scanned with Scn400 Image Viewer software (Leica, Solms, Germany). Tumors samples were screened at 10X magnification. The areas with a higher positive staining for CD31- as vascular hot spots- were further examined at 20X magnification to observe the vascular structures.

### Determination of clofazimine concentration in tissues

Clofazimine (10 nmol/g) was injected i.p. into control mice. 24 hours after last injection the animals were sacrificed and blood and various tissues were collected. Up to 100 mg of each tissue were weighted precisely, PBS (1 vol) was added, and the tissue sample was cut into small pieces and homogenized with an electric pestle. 100 μM 5-methoxypsoralen (5-MOP) in acetone (0.1 vol) was added as internal reference and clofazimine was then extracted adding 4.35 M acetic acid (0.1 vol) and acetone (10 vol). Each sample was vortexed (2 min), sonicated (2 min), and centrifuged (12,000 g, 7 min, 4°C); the supernatant was collected, concentrated using a Univapo 150H (Uniequip) vacuum concentrator, and finally analyzed via HPLC-UV according to established protocols previously used for other drugs [83]. Blood samples (80-100 μL) were obtained from the sacrificed animals and collected in heparinized tubes, kept in ice, and treated within 10 min. Blood treatment is was slightly different to from that applied to the organs: 100 μM 5-MOP (0.1 vol) was added as internal reference and clofazimine extracted adding 0.6 M acetic acid (0.1 vol) and acetone (4 vol). The samples were sonicated (2 min), and centrifuged (12,000 g, 7 min, 4°C) and then processed as just described. Recovery yields of clofazimine were determined as described in [83, 84]. HPLC analysis was performed with a 1290 Infinity LC system (Agilent Technologies) using a reverse phase column (Zorbax Extend-C18), 3.0 × 50mm, 1.8 micron, Agilent Technologies) and a UV diode array detector (190-500 nm). Solvents A and B were water containing 0.1% TFA (trifluoroacetic acid) and acetonitrile, respectively. The gradient for B was as follow: 10% for 0.5 min, then from 10 to 100% in 4.5 min, 100% for 1 min; the flow rate was 0.6 mL/min.
